# Pharmaceutical Policy Part 2 Pharmaceutical engagement and policy development: a framework for influence

**DOI:** 10.1186/s40545-015-0026-6

**Published:** 2015-02-10

**Authors:** Norman C Morrow

**Affiliations:** Commonwealth Pharmacists Association, 1 Lambeth High Street, London, SE1 7JN UK

**Keywords:** Pharmaceutical policy, Strategic planning, Medicines management, Pharmacists, Medication safety, Influencing

## Abstract

The formulation of pharmaceutical policy is a critical component of healthcare planning, made more important given that medicines are the ubiquitous technology in the diagnosis, treatment and prevention of disease and constitute a significant proportion of health care expenditure. Pharmacists need to inform policy development that will, in its implementation, offer opportunity to deliver greater rationality, safety, effectiveness and economy to the medicines use process and where patients experience enhanced health outcomes.

This is the second of two articles directed to this specific issue focusing on how policy and strategic change can be affected. This is discussed from three overlapping perspectives – from the point of view of skills, that is, the skills or tactics needed to be employed to effect change; secondly, from a structural standpoint in terms of what positional arrangements exist that could be positively exploited; and thirdly, the subject, particularly its relevance to the contemporary situation. These approaches are then exemplified through a worked example on medication safety and its application in practice.

## Background

Part 1 of this two part series commentary set out a number of practice challenges around pharmacy and medicines management, their implications for policy and the need for a balanced approach. It highlighted some key learning points in respect of formulating and implementing national medicines policies and cited a range of authoritative evidence sources to inform the development of pharmaceutical practice and medicines management policies. Emphasis was placed on the critical importance of the pharmaceutical profession to engage with national policy makers allied to the strategic planning for health care, as well as a commitment to measuring outcomes of pharmaceutical initiatives or interventions.

The conference report on the Asia Pacific Conference on National Medicines Policies made the following observation that sets a challenging context for influential engagement.

‘In a number of countries, the early gains in national medicines policy implementation have been lost due to a lack of ongoing political commitment and loss of policy champions. Governments need to recognise the importance of a strong legislative framework and a commitment to enforcing the law. Existing systems need to be strengthened and financial resources mobilised to address regional problems in providing access to medicines of appropriate quality, safety and efficacy. National medicines policies and activities to support them need to be presented as a compelling business case for governments to support and resource’ [1].

Engagement is, however, often easier said than done, especially if there has not been any history of involvement or where pharmaceutical considerations have been marginalised or even where pharmaceutical advice has been sourced from non-pharmacists. The question then becomes, ‘How is involvement and influence to be effected?’ Further, given success in initiating new actions, assessing processes and measuring outcomes will be crucial to future planning and potential new resource commitment. Again, this can prove a very challenging task, because of the complexities and cost of evaluation.

## Main text

**Figure 1:**
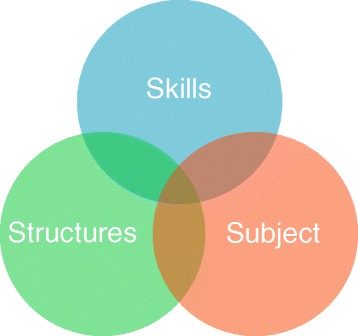
**Influencing perspectives.**

One way of addressing the engagement issue is to think of it from three overlapping perspectives (Figure 1) – from the point of view of skills, that is, the skills or tactics needed to be employed to effect change - the behavioural component; secondly, from a structural standpoint in terms of what positional arrangements exist that could be positively exploited - the environmental or organisational component; and thirdly, the subject, particularly its relevance to the contemporary situation - the content or thematic component.

### Influencing and persuading skills

This is probably best thought of in terms of influencing and persuading skills at the inter-professional interface, embracing, for example, health and social care professionals, policy makers and financial advisors. Health care systems have changed and are changing from those of exclusive professional domains to those where teamwork, multidisciplinary collaboration and integrated working offer greater benefit [2].

Such changes, however, create new demands and challenges for health professionals, not only in negotiating one’s own needs, roles and responsibilities but in understanding those of others and mindful of the interests of patients and the need to provide for their optimal care [3,4]. Being able to influence others and persuade them to a particular point of view or course of action will be critical to effective inter-professional communication, as it will to meaningful engagement in designing and delivering health care. The tactics used in influencing and persuading others can be summarised under the following nine categories [5]. In effect, they show how their application can offer a better chance of success but also how and why failure may have been experienced in the past.

### Power

Power may be expressed in six different ways. *Expert power* derives from being recognised as an expert in a particular field or possessing in-depth knowledge such that the credibility of expressed views is highly influential. *Referent power* can be used to associate ourselves, our views or practices, with those of other leading individuals and therefore give more credibility to our position, or elicit in others the desire to be similarly aligned. *Legitimate power* is a function of a hierarchical position, as distinct from the person and could, for example, reflect an employer-employee relationship. Being able to reward others if they comply with a request is an example of *reward power*, whereas *coercive power* stems from being able to enforce one’s will under threat of sanctions. Finally, *information power* is based on the content of the message such that a pharmacist publishing a safety notice following a number of adverse events is likely to influence subsequent practice.

### Threat/Fear

This tactic employs the use of fear arousing messages and the threat of adverse outcomes in order to gain compliance to a particular course of action. Such an approach is widely used in public health campaigns to warn against the dangers of, for example, smoking, sunburn, malaria or tuberculosis and designed to elicit protective behaviour. The evidence suggests that the success in using this tactic relies on three particular elements namely,

- the magnitude and perceived severity of the negative outcome;
- the probability of occurrence if no action is taken to avoid it; and
- the likely availability and effectiveness of the recommended course of action.

### Logical argument

Logical argument takes the form of reasoned appeal based on the presentation of the available evidence. It is not merely confined to the evidence itself but the conclusions emanating from it and the advantages of a particular course of action set against other alternatives. The power of the argument is further enhanced by the style of delivery, using slightly faster speech rate, an open posture, few hesitations or expressed doubts and the use of ‘intensifiers’ e.g. definitely, absolutely.

### Reciprocation

The maxim, ‘You scratch my back and I’ll scratch yours’ is an apt way to sum up this approach that is essentially an exchange of favours. It may entail giving something in advance (pre-giving) in anticipation of the favour being repaid later or it may involve a conditional promise, ‘You do this for me now, and I’ll do that for you later’.

### Moral appeals

This is an appeal to an individual’s sense of rightness and responsibility where acceptance is likely to lead to self-contentment, whereas rejection may induce feelings of guilt e.g. positively responding to an appeal for a charitable cause.

### Scale of request

This can take two forms: the ‘foot in the door’ or ‘door in the face’ strategies. The first involves a small scale request, ‘Could we try this new treatment in one or two patients’ but then escalating it to a higher demand e.g. a controlled clinical trial. The second could begin with the large demand but scaled down to make the request more moderate and appealing.

### Scarcity value

By rendering something as having limited or restricted availability and presented with constraints of time, finance, volume or uniqueness, induces pressure to gain early or immediate adoption. ‘if you can give approval for the programme within the next 2 weeks we can have the work completed within this current financial year’.

### The relationship

This approach derives from the fact that we are more likely to be influenced by people we like, are friendly with, or for whom we have a high regard. Thus the building and establishment of positive professional relationships will be important to effective persuasion attempts. Moreover, having supporters from other disciplines will improve the chances of success where there is seen to be an agreed position to a particular course of action.

### Aversive stimulation

An extreme example of this tactic is the torture of an individual in order to extract information. At an everyday level it is the persistent nagging of a parent by a child until the parent accedes. In work it is the continued and determined lobbying of management in anticipation of gaining a positive response that initially was not forthcoming.

### Structures

As indicated earlier, skills, structures and subject matter are not exclusive elements and it could be argued that structures, to some extent, form a part of the power dimension of influencing. On the one hand they reflect more organisational influence in that they often represent a sizeable body of opinion, have credibility among their peer organisations and are recognised by society at large, particularly those that have a professional regulatory role allied to public protection. On the other hand they may also reflect the systems or machinery of government for the ordering of society and which can be used as a vehicle through which influence can be exerted.

**Figure 2:**
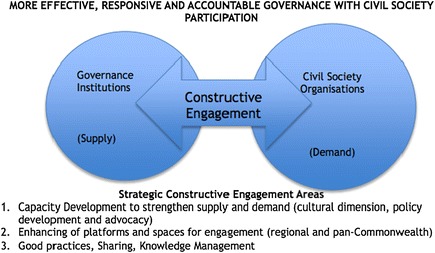
**Civil Society Organisations-Governance Constructive Engagement areas and Approaches [** 6 **].**

Although governments utilise the media to communicate with their populations at large, they may also engage with both national and local bodies that represent different interest groups. In some countries, like the UK, there are well established processes for public consultation that facilitate comment and expression of individual interests. Government consultation processes are therefore an opportunity for professional pharmaceutical bodies to make their voice heard in areas where they have a legitimate interest. Moreover, organisations like The Commonwealth Foundation seek to promote participatory governance among member countries (Figure 2) and indeed provide grant support for initiatives meeting its strategic objectives [6,7].

Outside a consultation process, representative bodies have the potential to lobby local politicians or Government. A number of national pharmaceutical bodies or associations have established a Public Affairs section to provide for a conduit between their organisation and public representatives. It is also important to appreciate the power of the media to shape public opinion and government policy and the influence that can be brought to bear through that route. At the same time pharmaceutical organisations that espouse standards of professional ethics allied to the delivery of pharmaceutical services need also to ensure that those professional ethics are consistently applied to their wider business.

Allied to engagement with policy makers, opportunities can be created by, for example extending an invitation to address a particular meeting or conference. This will provide the opportunity to offer some briefing setting out the roles of the organisation, the particular contributions of the profession as well as highlighting examples of innovative practice.

In the same way providing the opportunity for a public representative, government official or Minister to visit a particular practice site provides for further visibility of the profession and a setting in which pharmaceutical matters can be discussed and explored. In addition, it can be a good setting to elicit if a proposal or paper on a particular matter would be welcomed.

Structures would also embrace established forums, such as Review Groups, National Inquiries, Task Forces, Specialist Committees, created to investigate particular issues and make recommendations. In the health environment these are numerous, whether they concern the management of particular disease states, eg. management of long term conditions, making provision for elderly care, strategies to tackle communicable disease, inquiries into adverse events. These provide opportunity for pharmaceutical representation, but also importantly to express medicines management or pharmaceutical care perspectives and options. In summary.

- Are there professional organisational structures that allow for representation - national, regional, local?
- Are there media opportunities that enable pharmacy’s voice to be heard?
- Are there Review or Task Groups that would enable pharmaceutical involvement?
- Are there disease management or public health collaborative ventures that pharmacists can engage in?
- Where might the opportunity lie to invite a health care policy maker or health minister to address a meeting of pharmacists?
- Have you proposals at hand that could be readily offered given the opportunity to put them forward – ‘You don’t get a second chance to make a first impression!’
- Could you develop a proposal to support an engagement process on a particular matter of local, regional or national importance?

### Subject

Getting an issue or topic onto the policy agenda may prove particularly challenging and can require a good understanding of, and ability to, navigate the public policy arena [8,9], Moreover, in any given area there can be competing interests, for example, policies around rational prescribing of medicines and the pressure from pharmaceutical companies to expand the sales of their products [10]. However, the subject or the impact of the subject can itself be influential in ensuring that it has a prominent place in the healthcare policy agenda e.g. the risk of hospital acquired infection, requiring a policy to be developed or modified. Here it is the importance, urgency, exposure to avoidable risk, cost, public concern that is likely to influence action being taken.

These factors apply to medicines more generally in that they are the ubiquitous technology in health care, consume a considerable proportion of health expenditure, contribute substantially to the management and cure of disease but also are a recognised source of adverse effects due to their pharmacological properties and of adverse events due to errors in their use. They therefore deserve a prominent place in health care policy making. Some examples of medicine related policies are given in Table 1.

**Table 1:** **Examples of medicine related policies**

Adverse medication events	Prescription charges
Antimicrobial stewardship	Purchasing of medicines
Availability of high cost specialist medicines	Quality of Medicines
Essential medicine lists	Reimbursement of dispensed medication
Generic substitution	Storage, safe handling and administration
Medicines information for patients	Use of patients own medicines following hospital admission
Medicines legislative control	Use of unlicensed (and off-label) medicines
Partnership with the pharmaceutical industry	

### A worked example

How then might the significance of an issue for policy development be demonstrated? Consider, for example, adverse drug reactions (ADRs) that are often viewed as an inevitable risk of using medicines. National data may not be available but there is a considerable body of international research literature that indicates the level of risk particularly in the context of hospital care. While there is a range of values depending on the patient group, a conservative estimate would be that 4 – 6% of all hospital admissions are due to ADRs. The literature indicates that some 50 -70% of these are preventable. Assuming knowledge of the number of hospital admissions it is possible to work out the numbers likely to have been the result of an ADR. Data on the average length of stay would give an indication of the total bed days that could have been avoided and at what cost. The worked example shows the impact (Table 2).

**Table 2:** **Financial implications for ‘A Country’ in preventing hospitalisations due to adverse drug reactions**

Annual number of hospital admissions	500,000
No. of admissions at a 4 -6% ADR incidence level	20,000 - 30,000
At an average length of stay of 5 days	100,000 – 150,000
At an average cost of £300/day	£30 m - £45 m
Potential savings (based on 50%-70% preventability)	£15 m - £31.5 m

(NB Average length of stay and daily costs will be country specific).

Similarly, consideration of the published data on adverse events (as distinct from adverse drug reactions) in hospitalized patients this occurs in almost 10% of patients, and of these, some 10-20% are medication related [11]. Applying, therefore, a 1-2% incident level to the above scenario would yield the following savings (Table 3).

**Table 3:** **Financial implications for ‘A Country’ in preventing adverse medication events in hospitals**

Annual number of hospital admissions	500,000
No. of adverse medication events at a 1-2% level	5,000 - 10,000
At an average length of stay of 5 days	25,000 – 50,000
At an average cost of £300/day	£7.5 m - £15 m
Potential savings (based on 50%-70% preventability)	£3.75 m - £10.5 m

(NB Average length of stay and daily costs will be country specific).

However, the financial saving are not the only impact of improved medication practice. Consideration should also be given to the cost to the economy in terms of lost working days, the opportunity costs to provide other care, not to mention the human costs to the individual patient, family or carers.

Having then defined the problem, it is possible to begin to identify a potential range of options or solutions to minimize its impact and how, in particular, pharmaceutical expertise can be applied. Such solutions may include prescribing support, formulary development, patient adherence programmes, targeting of medications particularly associated with risk e.g. insulin, digoxin, anticoagulants, closer patient monitoring, reducing communication failures through, for example, improved documentation or information transfer.

This approach has proved successful, for example, in Northern Ireland leading to the establishment of a Government funded Pharmacist Governance team focused on minimising the occurrence of medication-related adverse events in hospital through a systems-based approach to risk management [12]. Built upon the success of the team and the contribution it has made to safer medication practice, the team has been extended into primary care [13,14]. Here the work has focused on:

- Encouraging the reporting of adverse incidents from GPs and Community pharmacists
- Establishing an anonymous adverse incident reporting system for community pharmacists
- Following up individual adverse incidents to ensure learning and reduce the chance of a similar adverse incident by the same practitioner
- Identifying and sharing learning from both named and anonymous adverse incidents across practitioners via regular newsletters and medicines safety alerts
- Developing Standard Operating procedures (SOPs), processes and audits for safer systems in general practice, e.g. repeat prescribing, prescription security, medication review
- Providing training to a range of audiences to promote adverse incident reporting, learning and implementation of safe systems
- Developing local learning resources and protocols for national alerts to ensure their implementation in primary care
- Developing systems and processes to oversee the safe management of Controlled Drugs in primary care

What has resulted is a whole systems approach to medication safety. This includes processes for handling medication incidents identified at the interface, development of medication incident categorisation to permit trend analysis across both sectors and the identification and collaboration on joint safety initiatives which support implementation of national (UK wide) safety alerts.

This is but one example of influencing for change but illustrative of the use and application of evidence to address a critical issue, particularly using pharmaceutical expertise. At a more macro level WHO has published a framework for the development and implementation of national drugs policies that can easily be adapted and applied to more specific topics such as those in Table 1 [15]. In addition, allied to the implementation of medicines policies, it is important to think about those who will have a key input to effecting their application and the degree to which their role needs to be consolidated or expanded. In that regard, pharmacists as regulated professionals, are a central resource both from an informational point of view but also as an integral member of the supply chain.

## Conclusion

This article has addressed three important components in respect of influencing pharmaceutical policy namely, the application of persuasive skills, the constructive use of existing structures to engage with policy makers and the identification of critical subject areas that, if addressed, could enhance the delivery of health care and lead to better health outcomes. Full engagement in these processes is important for pharmacists in order to fulfill their full potential as health care providers and more generally contribute to the welfare of society.
